# Two 4N Cell-Cycle Arrests Contribute to Cisplatin-Resistance

**DOI:** 10.1371/journal.pone.0059848

**Published:** 2013-04-01

**Authors:** Hong Shen, Ricardo E. Perez, Batzaya Davaadelger, Carl G. Maki

**Affiliations:** Department of Anatomy and Cell Biology, Rush University Medical Center, Chicago, Illinois, United States of America; University of Nebraska Medical Center, United States of America

## Abstract

Cisplatin is a platinum-based drug that is used for the treatment of a wide-variety of primary human cancers. However, the therapeutic efficacy of cisplatin is often limited by intrinsic or acquired drug resistance. An important goal, therefore, is to identify mechanisms that lead to cisplatin resistance in cancer, and then use this information to more effectively target resistant cells. Cisplatin-resistant clones of the HCT116 cell line underwent a prolonged G2 arrest after cisplatin treatment while sensitive clones did not. The staurosporine analog UCN-01 abrogated this G2 arrest and sensitized the resistant clones to cisplatin. At later time points, 4N arrested cells assumed a tetraploid G1 state that was characterized by depletion of Cyclin A, Cyclin B, and CDC2, and increased expression of p53 and p21, in 4N cells. siRNA-mediated knockdown of p21 abrogated the tetraploid G1 arrest and induced killing that was dependent on p53. The results identify two targetable 4N arrests that can contribute to cisplatin resistance: First, a prolonged G2 arrest that can be targeted by UCN-01, and second, a tetraploid G1 arrest that can be targeted by siRNA against p21.

## Introduction

Cisplatin (CP) is a platinum-based drug that is widely used in the treatment of various primary human cancers. CP induces DNA intra-strand and inter-strand crosslinks that can trigger cell cycle arrest, DNA repair, and/or apoptotic death [1]. CP has shown clinical efficacy against different cancer types, including testicular, ovarian, and head and neck cancer [1]. Nonetheless, the development of CP resistance remains a major obstacle to its clinical use. Thus, while tumors may show an initial killing response to CP and effectively be “cured”, they can often grow back in a form that is both therapy resistant and highly aggressive. It is therefore important to identify the molecular mechanisms that lead to CP resistance in cancer, and then use this information to target resistant cells.

The most prominent cell cycle responses to CP are an arrest or delay in S- and G2-phase [2], [3]. These arrests/delays are thought to allow time for DNA damaged cells to repair their DNA before proceeding with DNA synthesis or mitosis. The checkpoint kinases Chk1 and Chk2 are activated by CP and can play a role in the S- and G2-phase arrest/delay induced by CP [4]. Chk1 is activated by ATR in response to stalled replication forks in S-phase, and causes S-phase arrest/delay by inhibiting DNA replication origin firing [5]. Activated Chk1 and Chk2 can also promote a G2-phase arrest by phosphorylating and inactivating CDC25 phosphatase, and thus keeping the G2 phase cyclin dependent kinase CDC2 in a phosphorylated, inactive state [6]–[8]. Conceivably, abrogating these arrests may kill cancer cells by forcing them to reenter the cell cycle prematurely in the face of unrepaired DNA damage. With this goal in mind, various Chk1 and/or Chk2 inhibitors have been developed or are being developed for clinical use. UCN-01 is a broad range kinase inhibitor that can inhibit Chk1 and Chk2 (in addition to other kinases) and that has been tested in clinical cancer trials with chemotherapy and/or radiation [9], [10]. The ability of UCN-01 to abrogate G2-phase cell cycle arrest has been well-documented. Thus, UCN-01 was reported to abrogate the G2-phase arrest induced by either CP or ionizing radiation (IR), and to enhance CP- and IR-induced cancer cell killing [11], [12].

P53 is a tumor suppressor and key regulator of DNA damage responses. P53 is normally expressed at low levels due to a short protein half-life [13]–[15]. However, the p53 protein is stabilized and its levels increase in response various DNA damaging agents, including CP [16], [17]. Stabilized p53 can function as a transcription factor, inducing expression of various downstream genes that promote and/or regulate G1 or G2-phase cell cycle arrest, senescence, apoptosis, and metabolism [18]–[20]. P53 induces G1 arrest by inducing expression of p21, a cdk inhibitor that can bind G1 and S-phase cyclin-cdk complexes and inhibit their activity [21]. P53 induces or maintains a G2-phase arrest by inducing expression of various target genes, including *GADD45, P21,* and *14-3-3 σ,* which keep the G2-phase cyclin-B-CDC2 complex inactive [22]. Interestingly, cells that arrest in G2 for prolonged periods after DNA damage can sometimes undergo a process known as cell cycle adaptation, in which they reactivate CDC2 complexes and proceed with mitosis despite the presence of unrepaired, damaged DNA [23], [24]. This process most likely culminates in abortive mitotic attempts and cell death. Alternatively, prolonged and heightened p53-p21 signaling in G2-arrested cells may drive these cells into a G1-like state, referred to as tetraploid-G1, characterized by depletion/loss of G_2_/M marker proteins (*e.g.* Cyclins A/B, CDC2) and increased expression of G1-phase markers in 4N cells [25], [26]. In some cases, these tetraploid cells can enter S-phase, complete a division cycle, and survive with 4N DNA content [26]. P53-induced tetraploidy may be a survival mechanism that limits the formation of adaptive cells that would otherwise enter mitosis prematurely and die.

In this study, we isolated and expanded single cell clones from the HCT116 human colon cancer cell line, and compared their sensitivity to CP. Some clones were reproducibly CP resistant, while others were reproducibly CP-sensitive. To explore the basis for these differences, we monitored the cell cycle profiles of resistant and sensitive clones for extended periods of CP exposure. Both the sensitive and resistant clones displayed a G2 arrest when exposed to CP for relatively short periods. However, resistant clones appeared to maintain this G2 arrest while sensitive clones did not. UCN-01 abrogated the G2 arrest and sensitized resistant clones to CP. At later time points, CP treated cells arrested in a 4N, tetraploid G1- state that was p53 and p21-dependent and characterized by depletion of Cyclins A, B and CDC2. siRNA-mediated knockdown of p21 abrogated the tetraploid G1 arrest and induced killing that was dependent on p53. Together, these findings identify two targetable 4N arrests that can contribute to cisplatin resistance: First, a prolonged G2 arrest that can be targeted by UCN-01, and second, a tetraploid G1 arrest that can be targeted by siRNA against p21.

## Materials and Methods

### Cells and Reagents

HCT116 cells (described in [27]) were obtained from Dr. Bert Vogelstein (John Hopkins University) and were grown in McCoy's 5A medium with 10% fetal bovine serum (FBS), penicillin (100 U/mL) and streptomycin (100 µg/mL). Cells were plated 24h before being treated with Cisplatin (Bedford Laboratory) at the indicated concentrations. UCN-01 and colcemid were obtained from Sigma. UCN-01 was used at a final concentration of 500 nM, and colcemid was used at a final concentration of 10 ng/ml.

### Immunoblotting

Whole cell extracts were prepared by resuspending cell pellets in lysis buffer (150 mM NaCl, 5 mM EDTA, 0.5% NP40, 50 mM Tris, pH 7.5), resolved by sodium dodecyl sulfate polyacrylamide gel electrophoresis (SDS-PAGE) and transferred to polyvinylidene difluoride membranes (NEN Life Science Products). Antibodies to p21 (187), Cyclin A (H432), were from Santa Cruz Biotechnology; antibodies to Cyclin B1 (V152), CDC2 (POH1), p-CDC2 (Tyr15), Chk1 (2G1D5) and Chk2 (D9C6) were from Cell Signaling; antibodies to pRb (Ab-5) was from Calbiochem. Primary antibodies were detected with goat anti-mouse secondary antibodies conjugated to horseradish peroxidase (Jackson ImmunoResearch), using enhanced chemiluminescence (Perkin-Elmer).

### Flow Cytometry

For cell cycle analysis, cells were harvested and fixed in 25% ethanol overnight. The cells were then stained with propidium iodide (25 µg/mL, Calbiochem). For mitochondrial potential (ΛΨm) analysis, cells were harvested and stained with TMRE (Tetramethylrhodamine, Invitrogen, 0.1 µmol/L). For Annexin-V staining, cells were stained with Annexin V-PE and 7-amino-actinomycin D (7-AAD) (BD Pharmingen, San Diego, CA, USA) according to manufacturer’s instructions. Flow cytometry analysis was performed on FACS Canto (Becton Dickinson), analyzed with CellQuest (Becton Dickinson) and FlowJo 8.7 (Treestar Inc). For each sample, 10,000 events were collected.

### siRNA-mediated Transient Knock-down

p53, p21, Chk1, Chk2 RNAi (On-target plus smart pool) and Control RNAi (On-target plus siControl non-targeting pool) were purchased from Dharmacon and were transfected according to the manufacturer's guidelines using DharmaFECT I reagent.

## Results

### Identification of Cisplatin-sensitive and Cisplatin-resistant HCT116 Clones

**Figure 1:**
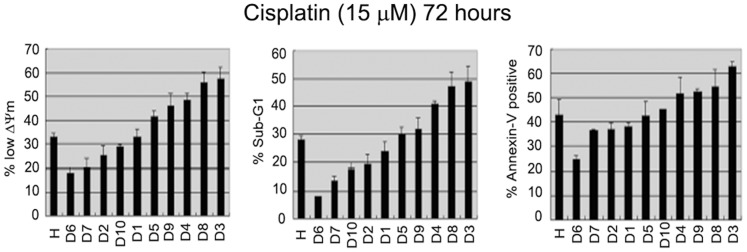
HCT116 clones vary in sensitivity to cisplatin. **A)** HCT116 cells were plated at single cell density and ten individual clones (designated D1–D10) were isolated and expanded. The clones were exposed to 15 µM Cisplatin for 72 hrs, and apoptosis determined by the percentage of cells with decreased mitochondrial membrane potential (% low ΛΨm), sub-G1 DNA content, or Annexin-V positive staining. (H) refers to the parental HCT116 cell population. Data represent the average of 3 experiments +/− s.e.m. **B)** Clones D3 and D6 were treated with 5 or 15 µM cisplatin for 24 hrs, followed by cisplatin removal. Percent colony formation was determined 2 weeks after cisplatin removal. Plotted is the average of 3 separate experiments +/− s.e.m.

HCT116 is a human colon cancer cell line that expresses wild-type p53. HCT116 cells were plated at single cell density and ten individual clones isolated (D1–10). Each clone was then treated with cisplatin, and apoptosis was monitored 72 hrs later by % sub-G1 cells, % Annexin-V positive cells, and % cells with decreased mitochondrial membrane potential. The HCT116 starting population (H) underwent approximately 30–40% apoptosis after CP treatment using all 3 apoptotic criteria (Fig. 1). Interestingly, individual clones displayed a wide range of apoptosis after CP that varied from relatively low to relatively high. For example, clones D6 and D7 reproducibly underwent ∼10–20% apoptosis after CP treatment, while other clones (e.g. D3 and D8) reproducibly underwent ∼50–60% apoptosis after CP treatment. It should be noted that the relative CP sensitivity in these clones appears to be a stable phenotype. Thus, sensitive and resistant clones maintained in culture for several months maintain their relative CP sensitivity (not shown). The results suggest HCT116 contains a mixture of clones that vary widely in their sensitivity to CP.

### CP-resistant HCT116 Clones Display a Prolonged 4N Arrest after CP Treatment

**Figure 2:**
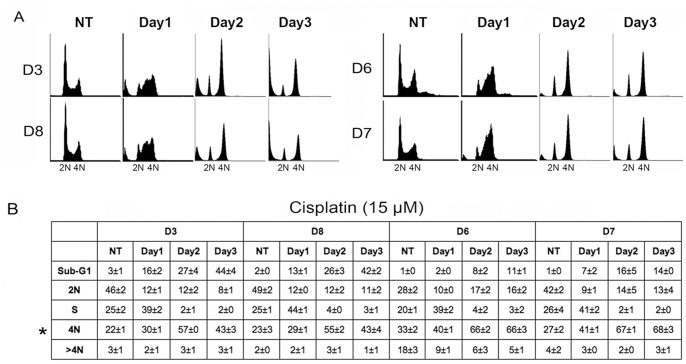
Cisplatin resistant HCT116 clones display a prolonged 4N arrest in response to cisplatin. **A)** HCT116 clones that are relatively cisplatin sensitive (D3, D8) or relatively cisplatin resistant (D6, D7) were untreated (NT) or treated with 15 µM cisplatin for 1–3 days. Cell cycle profiles were determined by flow cytometry. **B)** The percentage of sub-G1, 2N, S-phase, 4N, or greater than 4N cells was determined at the indicated time points in cisplatin treated cells. Numbers represent the average of 3 separate experiments +/− s.e.m.

The most prominent cell cycle responses to CP are arrests or delays in S- and G2-phase [2], [3]. We wished to investigate the basis for CP resistance and sensitivity in individual HCT116 clones. To this end, we selected two clones that were relatively CP sensitive (D3, D8) and two clones that were relatively CP resistant (D6, D7). We then monitored their cell cycle profiles when exposed to CP for 24–72 hrs (Fig. 2A, B). Both sensitive and resistant clones showed an initial accumulation/delay in S- and G2-phases at the 24 hr time point. At the 48 hr time point, both the sensitive and resistant clones displayed a loss of S-phase cells, and an accumulation of cells in a 4N state. D3 and D8 also began to show an accumulation of sub-G1 (apoptotic) cells at this 48 hr time point. Interestingly, at the 72 hr time point the CP-sensitive clones D3 and D8 displayed a loss (decreased percentage) of 4N arrested cells and a corresponding increase in the percentage of sub-G1 cells. In contrast, the percentage of 4N arrested cells was largely unchanged at the 48 and 72 hr time points in clones D6 and D7, and there was less sub-G1 death. These data suggest both sensitive and resistant clones undergo an initial S-phase delay followed by a 4N arrest after CP treatment. However, while resistant clones maintain this arrest to a large extent for up to 72 hrs, sensitive clones appear unable to maintain the arrest and die through sub-G1 apoptosis.

### UCN-01 Abrogates CP-induced G2 Arrest and Enhances Killing of CP-resistant HCT116 Clones

**Figure 3:**
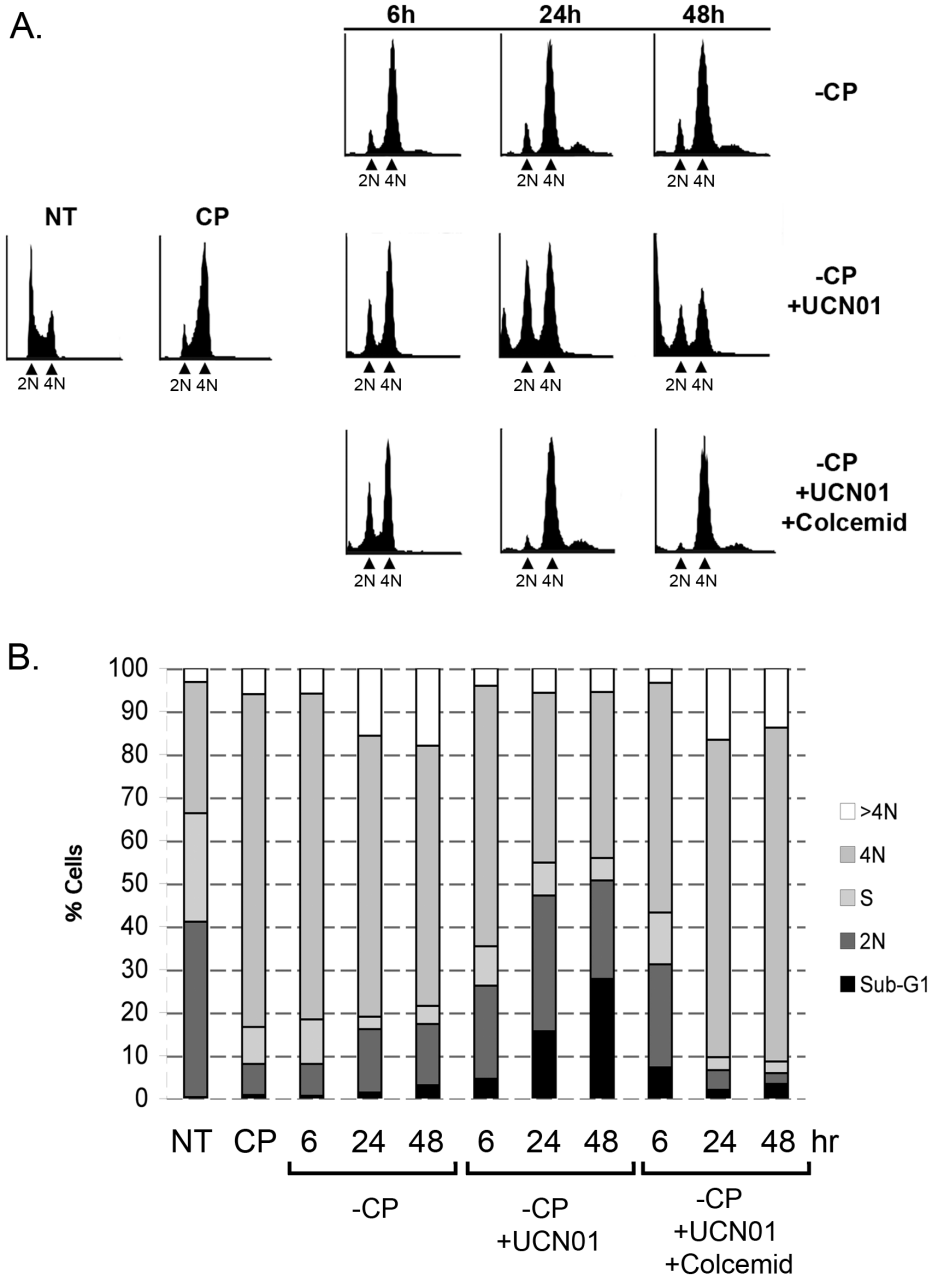
UCN01 abrogates CP-induced G2 arrest and enhances killing of CP-resistant HCT116. The CP resistant clone D6 was untreated (NT) or treated with 15 µM cisplatin for 6 hrs followed by cisplatin removal for 18 hrs (CP). The cisplatin was then removed by media change (−CP), and the cells were either untreated, treated with UCN01 alone, or treated with UCN01 plus colcemid for 6, 24, or 48 hrs. **A)** Cell cycle profiles were examined at the indicated time points. **B)** The percentage of sub-G1, 2N, S-phase, 4N, or greater than 4N cells was determined and is graphed.

UCN-01 is a staurosporine-derived anti-cancer agent that reversibly and ATP-competitively inhibits multiple protein kinases, including Chk1 and Chk2 [9], [10]. Previous studies showed UCN-01 could abrogate G2 arrest induced by either ionizing radiation or CP, and sensitize cells to IR or CP-induced killing (e.g. [11], [12]. We wished to test 1) whether UCN-01 could abrogate the apparent G2 arrest induced by CP, and 2) whether UCN-01 could sensitize the CP resistant clones to CP-induced killing. First, we used a mitotic trap assay to ask whether UCN-01 could abrogate a CP-induced G2 arrest. Briefly, the CP resistant clone D6 was treated with CP (15 µM) for 6 hrs and then cultured in the absence of CP for an additional 18 hrs. Under these conditions, the majority of cells were arrested in a 4N state (column marked CP in Fig. 3). The CP was then removed by media change, and the cells were then either untreated, treated with UCN01 alone (500 nM), or treated with UCN01 (500 nM) plus colcemid (10 ng/ml). We chose this UCN-01 dose based on previous studies that showed this dose inhibits Chk1 and sensitizes cells to CP [28], and our preliminary data which suggested this is the minimal dose that can fully inhibit Chk1 (not shown). Cell cycle profiles were examined 6, 24, and 48 hrs later. As shown in Figs. 3A and 3B, the majority of untreated cells remained arrested in a 4N state for up to 48 hrs after CP removal. In contrast, cells treated with UCN-01 alone (−CP +UCN01) showed a decrease in the amount of 4N arrested cells with an increase in both 2N cells and sub-G1 cells at the 24 hr and 48 hr time points. This suggested that UCN-01 was causing 4N arrested cells to enter mitosis and divide, and that some of these prematurely dividing cells were dying and accumulating in a sub-G1 state. Colcemid is a microtubule inhibitor that traps cells in mitosis by blocking chromosome segregation. We reasoned that if UCN-01 was killing cells by forcing them into mitosis, then cells treated with UCN-01 plus colcemid would remain arrested in a 4N state. Consistent with this, we observed that cells treated with UCN-01 plus colcemid after CP removal (−CP+UCN01+colcemid) remained arrested in a 4N state for up to 48 hrs, apparently having been trapped in mitosis (Figs. 3A and 3B).

**Figure 4:**
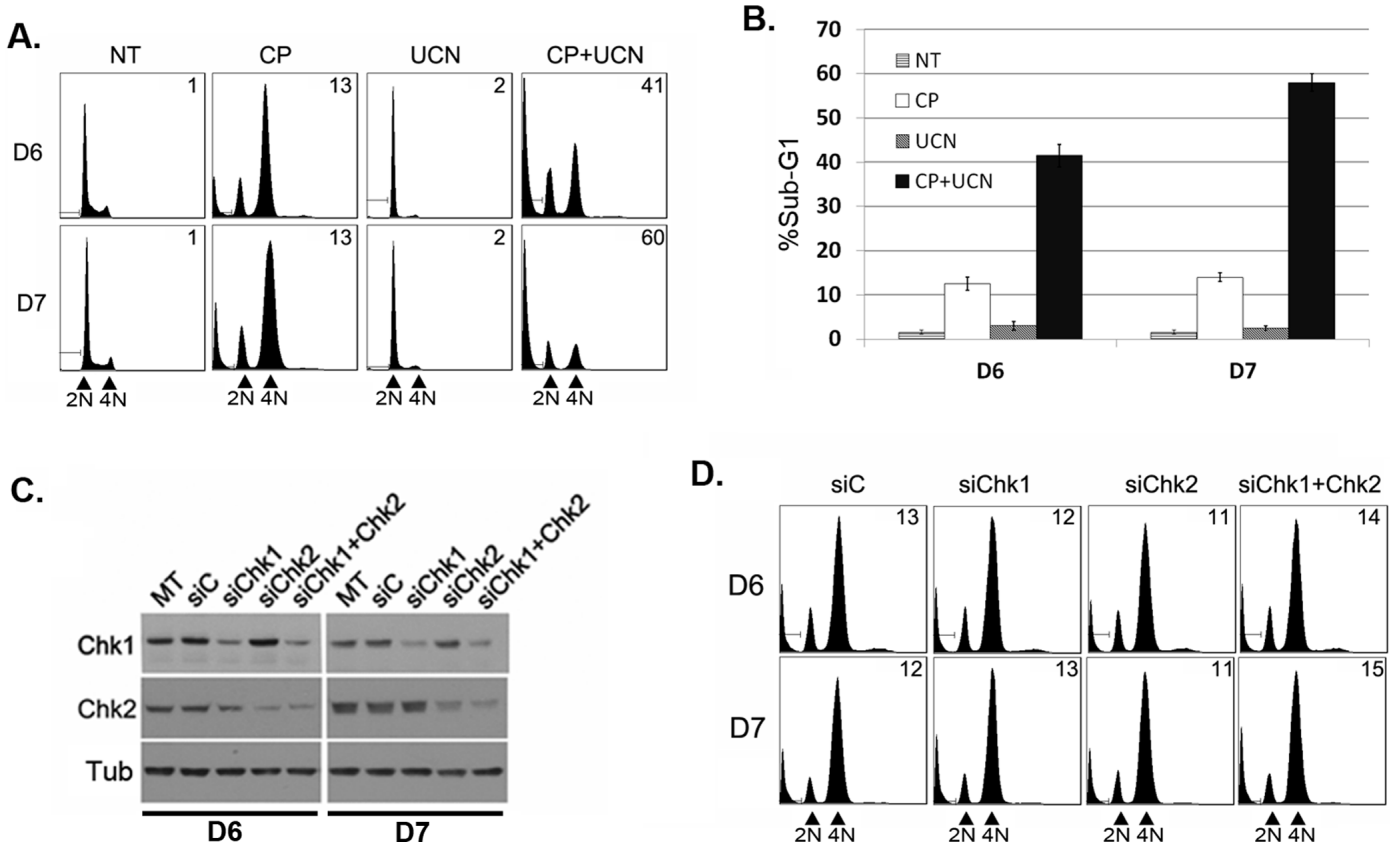
UCN01 sensitizes CP-resistant HCT116 clones to CP-induced killing. HCT116 clones D6 and D7 were treated with either CP alone for 72 hrs, UCN01 alone for 72 hrs, or treated with CP alone for 24 hrs and then treated with UCN01 in the continued presence of CP for an additional 48 hrs. **A)** Cell cycle analysis was determined at the 72 hr time point. Representative cell cycle profiles are shown. The number in the upper right corner is the percentage of sub-G1 cells. **B)** Plotted is the percentage of sub-G1 (apoptotic) cells at the 72 hr time point. The data represent the average of 3 experiments +/− s.e.m. **C)** Clones D6 and D7 were either mock-transfected (MT) or transfected with control siRNA (siC) or siRNA against Chk1 and/or Chk2. Immunoblotting was performed 24 hrs after transfection. **D)** Clones D6 and D7 were transfected with the indicated siRNA for 24 hrs, followed by cisplatin (15 µM) treatment for an additional 72 hrs. Representative cell cycle profiles are shown. The number in the upper right corner is the percentage of sub-G1 (apoptotic) cells.

Next we asked whether UCN-01 could sensitize CP-resistant HCT116 cells to CP-induced killing. HCT116 clones D6 and D7 were treated with either CP alone for 72 hrs, UCN-01 alone for 72 hrs, or treated with CP alone for 24 hrs and then treated with UCN-01 in the continued presence of CP for an additional 48 hrs. Apoptosis (% sub-G1 cells) was monitored at the 72 hr time point. As shown in Fig. 4A and B, UCN-01 alone failed to induce apoptosis in clones D6 and D7, and CP alone also caused little apoptosis in these cells (10–15% sub-G1). In contrast, an abundant increase in sub-G1 cells was observed when clones D6 and D7 were treated sequentially with CP for 24 hrs, followed by the addition of UCN-01 for an additional 48 hrs (CP+UCN, Figs. 4A and B). The results demonstrate UCN-01 abrogates a CP-induced G2 arrest (Fig. 3), and enhances CP-induced killing in CP resistant HCT116 clones (Figs. 4A and B).

### Knockdown of Chk1 or Chk2 does not Mimic the Effects of UCN01

Chk1 and Chk2 are reported to play important roles in the S- and G2-phase arrests induced by DNA damaging stress, and UCN-01 can inhibit Chk1 and Chk2 activity [11], [12], [29]. We reasoned that if UCN-01 enhances CP-induced killing by inhibiting Chk1 or Chk2, then knockdown of Chk1 or Chk2 should mimic the effects of UCN-01 and also enhance killing by CP. To test this, CP resistant clones D6 and D7 were transfected with siRNAs to knockdown Chk1 and/or Chk2, and then examined for CP sensitivity. Immunoblotting (Fig. 4C) showed a pronounced reduction in Chk1 and Chk2 protein levels in the siRNA transfectants. Despite this, however, the cells remained resistant to CP-induced killing (Fig. 4D). While Chk1 and Chk2 knockdown was not complete (there was still residual Chk1 and Chk2 expressed in the siRNA transfectants), the results nonetheless suggest that Chk1/Chk2 knockdown does not mimic the effects of UCN-01.

### Prolonged CP Treatment Induces a Tetraploid G1 Arrest that is p53- and p21-dependent

**Figure 5:**
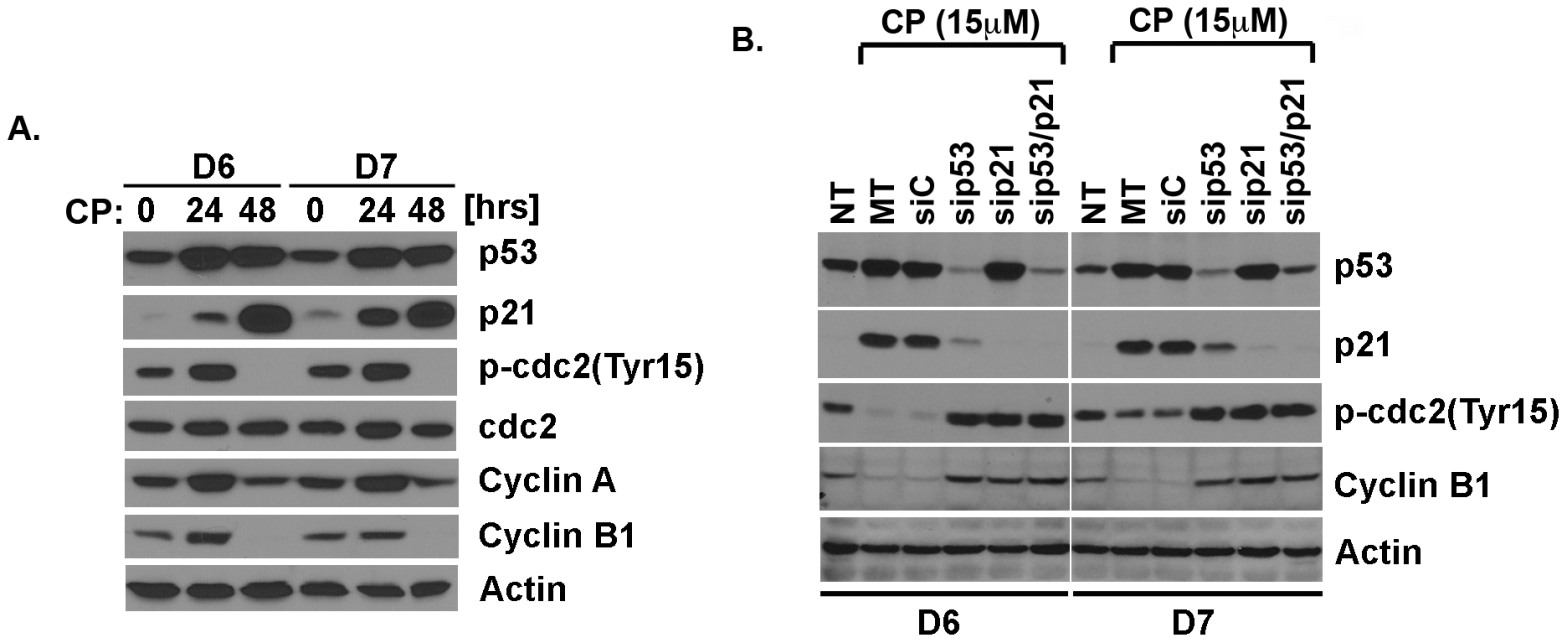
Cisplatin induces a tetraploid G1 arrest that is p53 and p21 dependent. **A)** HCT116 clones D6 and D7 were treated with 15 µM cisplatin (CP) for 24 or 48 hrs, followed by immunoblotting for the indicated proteins. Actin levels were used as a loading control. **B)** HCT116 clones D6 and D7 mock transfected (MT) or transfected with control siRNA or siRNA targeting p53 or p21. Twenty four hrs after transfection, the cells were treated with cisplatin (15 µM) for an additional 48 hrs. Protein lysates examined by immunoblotting.

Cells that are G2 arrested for prolonged periods can enter a G1-like state, referred to as tetraploid G1 arrest [25], [26]. This tetraploid G1 arrest is characterized by decreased expression of Cyclin B, Cyclin A, and/or CDC2, such that a condition of low CDK activity resembling early G1 phase is established in 4N cells. To investigate whether CP treated and 4N arrested cells were in a tetraploid G1 state, we compared levels of Cyclin B1, Cyclin A, and CDC2 in cells that were CP treated for 24 or 48 hrs. Cyclins A and B1 accumulate during the cell cycle and are high in G2 phase, but then decrease rapidly as cells progress through mitosis. DNA damaging agents can arrest cells in G2-phase, in part, through inhibitory phosphorylation of CDC2 at tyrosine-15 (Tyr-15). D6 and D7 cells that were CP treated for 24 hrs expressed Tyr-15 phosphorylated CDC2 (pCDC2) and elevated levels of Cyclins A and B, consistent with being arrested in G2 phase (Fig. 5A). In contrast, cells that were CP treated for 48 hrs expressed low/undetectable levels of Cyclin B1, diminished levels of Cyclin A, and low/undetectable levels of pCDC2 (Fig. 5A). Total levels of CDC2 were also modestly decreased at this 48 hr time point. Previous studies reported that prolonged p53-p21 pathway activation can cause depletion of Cyclins A/B, and pCDC2 and promote a tetraploid G1 arrest [25], [26]. Consistent with this, p21 was induced after CP treatment in D6 and D7 at the 24 hr time point, but induced to a much higher level at the 48 hr time point when depletion of Cyclins A, B, and pCDC2 was observed (Fig. 5A). P53 was also induced at the 24 and 48 hr time points. The results indicate D6 and D7 cells that were CP treated for 48 hrs were undergoing a tetraploid G1 arrest. Next, we examined whether p53 and/or p21 are required for downregulation of Cyclin A, Cyclin B, and/or pCDC2 in CP-treated cells. Clones D6 and D7 transfected with control siRNA or siRNA targeting p53 or p21 were CP treated for 48 hrs, and protein lysates examined by immunoblotting. Cyclins A and B, and pCDC2 were markedly decreased in control siRNA transfected cells, but not in cells with either p53 or p21 knocked down (Fig. 5B). These results indicate p53-p21 pathway activation is required for decreased expression of Cyclin A, Cyclin B1, and pCDC2 in the CP treated cells.

**Figure 6:**
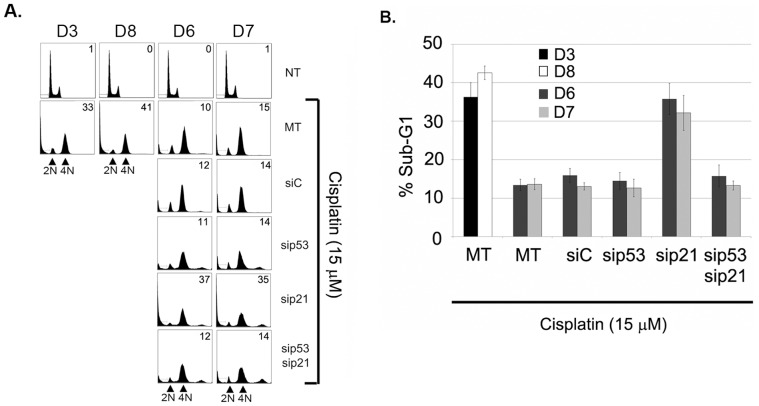
P21 knockdown abrogates the tetraploid G1 arrest induced by cisplatin and sensitizes cells to p53-dependent killing. **A)** HCT116 clones were either untreated (NT) or treated with 15 µM cisplatin. At the 24 hr time point after cisplatin treatment, cells were either mock transfected (MT) or transfected with the indicated siRNAs, and cultured in the continued presence of cisplatin for an additional 48 hrs. Representative cell cycle profiles are shown. The number in the upper right corner is the percentage of sub-G1 cells. **B)** Plotted is the percentage of sub-G1 (apoptotic) cells from the experiments in A). The data represent the average of 3 experiments +/− s.e.m.

Finally, we asked whether the tetraploid G1 arrest induced by p53 and p21 was protecting the cells from CP-induced killing. To this end, we used siRNA to knock down p53 or p21 in D6 and D7 cells that had been CP treated for 24 hrs. Cell cycle profiles and apoptosis (sub-G1 cells) were monitored at the 72 hr time point. As shown in Fig. 6A and B, the D6 and D7 cells that were mock transfected (MT) or transfected with control siRNA accumulated in a 4N state when treated with CP and were largely resistant to CP induced killing. In contrast, with p53 knockdown there was a decrease in 4N cells and a corresponding increase in cells with greater than 4N DNA content (Fig. 6A). This is consistent with knockdown of p53 abrogating the tetraploid G1 arrest and allowing some 4N G1-phase cells to enter S-phase and resume cycling. Notably, p53 knockdown cells remained resistant to CP-induced killing (Fig. 6A and 6B). With p21 knockdown there was also a decrease in 4N cells and an increase in greater than 4N cells, indicating that the tetraploid G1 arrest was abrogated (Fig. 6A). In contrast to p53 knockdown, however, the p21 knockdown cells were sensitized to CP (Fig. 6A and 6B). Finally, we used siRNA targeting to ask whether death in the p21 knockdown cells was p53-dependent. As shown in Figs. 6A and 6B, cells with simultaneous knockdown of p53 and p21 were no longer CP sensitive. Based on these results, we conclude 1) that prolonged (48 hr) CP treatment promotes a tetraploid G1 arrest in clones D6 and D7 that is p53 and p21-dependent, and 2) that knockdown of p21 abrogates the tetraploid G1 arrest and sensitizes CP treated cells to p53-dependent killing.

## Discussion

Cisplatin is commonly used in the treatment of various human cancers, including testicular, ovarian, osteosarcoma, and head and neck cancer. In most cases it is used in combination with other anticancer drugs. However, the clinical effectiveness of cisplatin is often limited by an intrinsic or acquired resistance to the drug. An important and long-term goal, therefore, is to establish the mechanisms responsible for cisplatin resistance in cancer, and then use this information to more effectively target resistant cells. In the current study, we compared cisplatin responses at multiple time points in clones of the HCT116 human colon cancer cell line. At early time points, CP resistant clones appeared to undergo a prolonged G2-phase arrest while CP sensitive clones did not. UCN-01 (7-hydroxystaurosporine) could abrogate this apparent G2-phase arrest and sensitize the resistant cells to cisplatin. At later time points, G2-phase (4N) cells entered a tetraploid G1 arrest that was p53 and p21-dependent, and characterized by depletion of G2-phase arrest marker proteins (Cyclins A and B, pCDC2) in 4N cells. Knockdown of p21 (via siRNA) released cells from the tetraploid G1 arrest, and the cells died in a p53-dependent manner. Together, these data indicate that 1) a prolonged G2 phase arrest and/or a tetraploid G1 arrest can contribute to cisplatin resistance in HCT116 cells, and 2) that abrogating these arrests by either UCN-01 (G2 arreest) or p21 knockdown (tetraploid G1 arrest) can enhance cisplatin sensitivity.

DNA damage can trigger cell cycle arrests throughout the cell cycle. One function of these arrests is to allow DNA damaged cells time to repair their DNA before proceeding with cell division, thus increasing survival (e.g. [6], [30]. In our studies, CP resistant HCT116 clones appeared to undergo a prolonged G2-phase arrest after CP treatment while CP sensitive clones did not. Importantly, UCN-01 treatment abrogated this G2-phase arrest and sensitized the resistant cells to cisplatin. This is consistent with the notion that prolonged G2 arrest contributed to cisplatin resistance. Studies by Eastman and colleagues are consistent with our results and also showed UCN-01 could abrogate G2 arrest and enhance cisplatin-induced killing [12]. Current models suggest Chk1 and Chk2 can promote or maintain G2 arrest by inhibiting the CDC25 phosphatase and thus maintaining CDC2 in a phosphorylated, inactive state [22]. Since UCN-01 can inhibit Chk1 and Chk2, we speculated that the sensitization effect of UCN-01 might result from Chk1 or Chk2 inhibition. However, we found that Chk1 and/or Chk2 knockdown was unable to sensitize the resistant cells to cisplatin. One possibility is that the residual Chk1 and Chk2 expressed in the knockdown cells was sufficient to maintain G2 arrest upon UCN-01 treatment. Alternatively, sensitization by UCN-01 may occur through mechanisms other than inhibiting Chk1 or Chk2. In this regard, it is important to note that UCN-01 may inhibit other cell cycle and apoptosis-related kinases when used at elevated doses, such as cyclin-dependent kinases (CDKs) [31], protein kinase C (PKC) [32], phosphoinositide dependent kinase 1 (PDK1) [33], and AKT [33], [34]. The sensitization to CP that we observe with UCN-01 treatment could result from inhibition of these or other kinases.

An interesting finding from the current study was that prolonged CP treatment induced a tetraploid G1 arrest in HCT116 clones. This tetraploid G1 arrest was characterized by high levels of G1 arrest marker proteins (p53 and p21), and depletion of G2-phase arrest markers (Cyclin A, Cyclin B, pCDC2), in 4N cells. Depletion of G2 marker proteins in cisplatin treated cells was p53 and p21-dependent. Moreover, knockdown of p53 or p21 after CP treatment caused a decrease in the amount of 4N arrested cells, and the appearance of cells with >4N DNA content, consistent with 4N cells entering S-phase. Together, these results suggest that p53 and p21 were required for and promoted a tetraploid G1 arrest in cisplatin-resistant HCT116 cells. Finally, p21 knockdown in cisplatin-arrested 4N cells resulted in increased cell death that could be rescued by simultaneous knockdown of p53. Based on this, it appears that a tetraploid G1 arrest can contribute to cisplatin resistance in HCT116 cells, and that abrogation of this arrest by p21 knockdown targets cells for p53-dependent death.
